# Dimethyl 1-cyano­methyl-1*H*-pyrazole-3,5-dicarboxyl­ate

**DOI:** 10.1107/S160053680902306X

**Published:** 2009-06-20

**Authors:** Zhi-Rong Qu

**Affiliations:** a Ordered Matter Science Research Center, College of Chemistry and Chemical Engineering, Southeast University, Nanjing 210096, People’s Republic of China

## Abstract

The title mol­ecule, C_9_H_9_N_3_O_4_, syhthesized from 1*H*-pyrazole-3,5-dicarboxylic acid and 2-bromo­acetonitrile, is approximately planar; the inter­planar angles between the pyrazole ring and the mean planes of the two carboxylate units and the cyanomethyl unit are 4.49 (10), 5.56 (9) and 5.03 (19)°, respectively. In the crystal, inversion dimers linked by pairs of weak C—H ⋯O bonds occur, and the packing is further stabilized by aromatic π–π stacking [centroid–centroid separation = 3.793 (4) Å].

## Related literature

For details of the preparation of nitrile compounds, see: Lee *et al.*(1989 ▶); Chambers *et al.* (1985 ▶). For the chemistry of pyrazole-related compounds, see: Radl *et al.* (2000 ▶); Dai *et al.* (2008 ▶); Fu *et al.* (2007 ▶); Xiao *et al.* (2008 ▶).
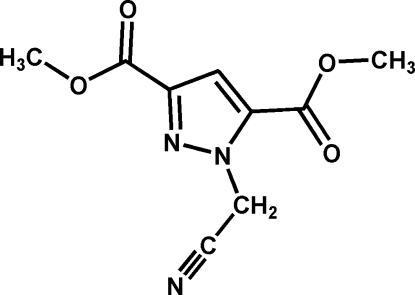


## Experimental

### 

#### Crystal data

C_9_H_9_N_3_O_4_
*M* *_r_* = 223.19
Triclinic, 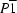

*a* = 6.865 (6) Å
*b* = 7.779 (7) Å
*c* = 11.133 (11) Å
α = 71.633 (8)°
β = 80.625 (10)°
γ = 68.195 (6)°
*V* = 523.2 (8) Å^3^
*Z* = 2
Mo *K*α radiation
μ = 0.11 mm^−1^
*T* = 293 K
0.25 × 0.17 × 0.15 mm

#### Data collection

Rigaku SCXmini diffractometer
Absorption correction: multi-scan (*CrystalClear*; Rigaku, 2005 ▶) *T*_min_ = 0.977, *T*_max_ = 0.983
5303 measured reflections
2356 independent reflections
1363 reflections with *I* > 2σ(*I*)
*R*_int_ = 0.042

#### Refinement

*R*[*F* ^2^ > 2σ(*F* ^2^)] = 0.063
*wR*(*F* ^2^) = 0.177
*S* = 1.07
2356 reflections
147 parameters
H-atom parameters constrained
Δρ_max_ = 0.14 e Å^−3^
Δρ_min_ = −0.25 e Å^−3^

### 

Data collection: *CrystalClear* (Rigaku, 2005 ▶); cell refinement: *CrystalClear*; data reduction: *CrystalClear*; program(s) used to solve structure: *SHELXS97* (Sheldrick, 2008 ▶); program(s) used to refine structure: *SHELXL97* (Sheldrick, 2008 ▶); molecular graphics: *SHELXTL* (Sheldrick, 2008 ▶); software used to prepare material for publication: *PRPKAPPA* (Ferguson, 1999 ▶).

## Supplementary Material

Crystal structure: contains datablocks I, global. DOI: 10.1107/S160053680902306X/kp2222sup1.cif

Structure factors: contains datablocks I. DOI: 10.1107/S160053680902306X/kp2222Isup2.hkl

Additional supplementary materials:  crystallographic information; 3D view; checkCIF report

## Figures and Tables

**Table 1:** Hydrogen-bond geometry (Å, °)

*D*—H⋯*A*	*D*—H	H⋯*A*	*D*⋯*A*	*D*—H⋯*A*
C2—H2⋯O3^i^	0.93	2.33	3.256 (4)	176

Symmetry code: (i) 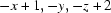.

**Fig. 1.**
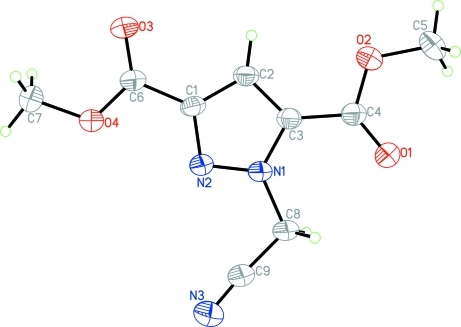
The molecular structure of the title compound with the displacement ellipsoids drawn at the 30% probability level.

**Fig. 2.**
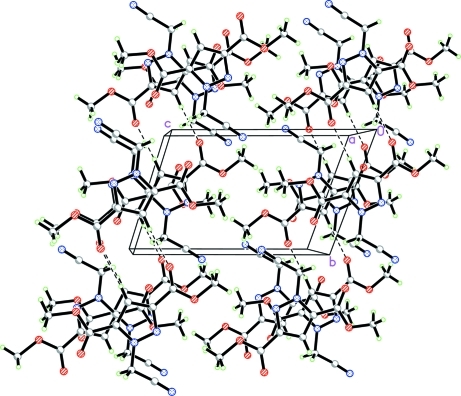
Packing diagram of the title compound, showing the structure along the b axis. Hydrogen bonds are shown as dashed lines.

## supplementary crystallographic information

### Comment

Pyrazole-related molecules have attracted considerable attention due to their biological activities (Lee *et al.*, 1989; Chambers *et al.*, 1985). In addition, the nitrile derivatives are important materials in the synthesis of some heterocyclic molecules (Radl *et al.*, 2000). We have reported many nitrile compounds (Dai *et al.*, 2008; Fu *et al.*, 2007; Xiao *et al.*, 2008). Here we report another nitrile compound, which was prepared from 1*H*-pyrazole-3,5-dicarboxylate and 2-bromoacetonitrile.

The title molecule, C_9_H_9_N_3_O_4_, syhthesized from 1*H*-pyrazole-3,5-dicarboxylate and 2-bromoacetonitrile, is nearly planar; the interplanar angles between the pyrazole ring and the mean planes of the carboxlate units and the acetonitrile unit are 4.49 (10), 5.56 (9) and 5.03 (19) respectively. No classical hydrogen bonds were found, but the weak hydrogen bond C2—H2 ···O3 (Table 1) connects molecule into a linear chain, and the structure is stablized by π-π stacking interactions [3.793 (4) Å] between the neighbouring pyrazole rings. (Table 2).

### Experimental

1*H*-pyrazole-3,5-dicarboxylic acid dimethyl ester (0.185 mg, 1 mmol) and 2-bromoacetonitrile (0.119 mg,1 mmol) were dissolved in acetone in the presence of K_2_CO_3_ (0.138 mg,1 mmol) and heated to reflux for 1 day. After the mixture was cooled to room temperature, the solution was filtered and the solvents removed in vacuum to afford a white precipitate of the title compound. Colourless crystals suitable for X-ray diffraction were obtained from a solution of 100 mg in 15 ml diethylether by slow evaporation after 7 days.

### Refinement

Positional parameters of all the H atoms were calculated geometrically and were allowed to ride on the C atoms to which they are bonded, with C—H = 0.93 Å (aromatic), 0.97 Å (methylene) or 0.96 Å (methyl) with *U*_iso_(H) = 1.2*U*_eq_(Caromatic, Cmethylene) or *U*_iso_(H) = 1.5*U*_eq_(Cmethyl).

### Crystal data

**Table:**

C_9_H_9_N_3_O_4_	*V* = 523.2 (8) Å^3^
*M**_r_* = 223.19	*Z* = 2
Triclinic, *P*1	*F*(000) = 232
*a* = 6.865 (6) Å	*D*_x_ = 1.417 Mg m^−^^3^
*b* = 7.779 (7) Å	Mo *K*α radiation, λ = 0.71073 Å
*c* = 11.133 (11) Å	µ = 0.11 mm^−^^1^
α = 71.633 (8)°	*T* = 293 K
β = 80.625 (10)°	Prism, colourless
γ = 68.195 (6)°	0.25 × 0.17 × 0.15 mm

### Data collection

**Table:**

Rigaku SCXmini diffractometer	2356 independent reflections
Radiation source: fine-focus sealed tube	1363 reflections with *I* > 2σ(*I*)
graphite	*R*_int_ = 0.042
CCD_Profile_fitting scans	θ_max_ = 27.5°, θ_min_ = 2.9°
Absorption correction: multi-scan (*CrystalClear*; Rigaku, 2005)	*h* = −8→8
*T*_min_ = 0.977, *T*_max_ = 0.983	*k* = −10→10
5303 measured reflections	*l* = −14→14

### Refinement

**Table:**

Refinement on *F*^2^	Primary atom site location: structure-invariant direct methods
Least-squares matrix: full	Secondary atom site location: difference Fourier map
*R*[*F*^2^ > 2σ(*F*^2^)] = 0.063	Hydrogen site location: inferred from neighbouring sites
*wR*(*F*^2^) = 0.177	H-atom parameters constrained
*S* = 1.07	*w* = 1/[σ^2^(*F*_o_^2^) + (0.0798*P*)^2^ + 0.0028*P*] where *P* = (*F*_o_^2^ + 2*F*_c_^2^)/3
2356 reflections	(Δ/σ)_max_ < 0.001
147 parameters	Δρ_max_ = 0.14 e Å^−^^3^
0 restraints	Δρ_min_ = −0.25 e Å^−^^3^

### Special details

**Table:**

Geometry. All e.s.d.'s (except the e.s.d. in the dihedral angle between two l.s. planes) are estimated using the full covariance matrix. The cell e.s.d.'s are taken into account individually in the estimation of e.s.d.'s in distances, angles and torsion angles; correlations between e.s.d.'s in cell parameters are only used when they are defined by crystal symmetry. An approximate (isotropic) treatment of cell e.s.d.'s is used for estimating e.s.d.'s involving l.s. planes.
Refinement. Refinement of *F*^2^ against ALL reflections. The weighted *R*-factor *wR* and goodness of fit *S* are based on *F*^2^, conventional *R*-factors *R* are based on *F*, with *F* set to zero for negative *F*^2^. The threshold expression of *F*^2^ > σ(*F*^2^) is used only for calculating *R*-factors(gt) *etc*. and is not relevant to the choice of reflections for refinement. *R*-factors based on *F*^2^ are statistically about twice as large as those based on *F*, and *R*- factors based on ALL data will be even larger.

### Fractional atomic coordinates and isotropic or equivalent isotropic displacement parameters (Å^2^)

**Table:**

	*x*	*y*	*z*	*U*_iso_*/*U*_eq_	
C1	0.3907 (4)	0.3854 (3)	0.8442 (2)	0.0532 (6)	
C2	0.3416 (4)	0.3412 (3)	0.9739 (2)	0.0526 (6)	
H2	0.3711	0.2192	1.0311	0.063*	
C3	0.2401 (4)	0.5159 (3)	1.0001 (2)	0.0499 (6)	
C4	0.1549 (4)	0.5643 (4)	1.1201 (2)	0.0546 (6)	
C5	0.1088 (6)	0.4259 (5)	1.3408 (3)	0.0854 (10)	
H5A	0.2016	0.4738	1.3658	0.128*	
H5B	0.1149	0.3033	1.3989	0.128*	
H5C	−0.0322	0.5153	1.3414	0.128*	
C6	0.5019 (4)	0.2473 (4)	0.7684 (3)	0.0598 (7)	
C7	0.6214 (6)	0.2060 (4)	0.5643 (3)	0.0843 (10)	
H7A	0.7659	0.1417	0.5851	0.126*	
H7B	0.6136	0.2814	0.4773	0.126*	
H7C	0.5551	0.1121	0.5772	0.126*	
C8	0.1385 (5)	0.8634 (3)	0.8624 (3)	0.0684 (8)	
H8A	−0.0104	0.8971	0.8865	0.082*	
H8B	0.2023	0.9057	0.9142	0.082*	
C9	0.1666 (5)	0.9611 (4)	0.7305 (3)	0.0727 (8)	
N1	0.2333 (3)	0.6541 (3)	0.88623 (19)	0.0519 (5)	
N2	0.3246 (3)	0.5768 (3)	0.7904 (2)	0.0554 (6)	
N3	0.1808 (6)	1.0496 (4)	0.6284 (3)	0.1128 (12)	
O1	0.0817 (3)	0.7247 (3)	1.13202 (17)	0.0727 (6)	
O2	0.1727 (3)	0.4040 (3)	1.21396 (18)	0.0693 (6)	
O3	0.5740 (4)	0.0765 (3)	0.8165 (2)	0.0895 (8)	
O4	0.5140 (3)	0.3323 (2)	0.64578 (17)	0.0697 (6)	

### Atomic displacement parameters (Å^2^)

**Table:**

	*U*^11^	*U*^22^	*U*^33^	*U*^12^	*U*^13^	*U*^23^
C1	0.0572 (15)	0.0334 (12)	0.0590 (16)	−0.0092 (11)	−0.0024 (12)	−0.0072 (11)
C2	0.0561 (15)	0.0386 (13)	0.0536 (16)	−0.0145 (12)	−0.0002 (12)	−0.0036 (11)
C3	0.0509 (14)	0.0420 (14)	0.0503 (15)	−0.0140 (11)	−0.0028 (11)	−0.0062 (11)
C4	0.0588 (16)	0.0464 (15)	0.0551 (16)	−0.0175 (12)	−0.0031 (12)	−0.0099 (12)
C5	0.114 (3)	0.086 (2)	0.0453 (17)	−0.033 (2)	0.0022 (16)	−0.0077 (15)
C6	0.0692 (18)	0.0396 (14)	0.0589 (17)	−0.0130 (13)	−0.0013 (13)	−0.0057 (12)
C7	0.110 (3)	0.0609 (19)	0.0632 (19)	−0.0110 (17)	0.0130 (17)	−0.0222 (15)
C8	0.090 (2)	0.0368 (13)	0.0594 (18)	−0.0099 (14)	−0.0006 (15)	−0.0041 (12)
C9	0.093 (2)	0.0416 (14)	0.066 (2)	−0.0115 (14)	0.0034 (16)	−0.0100 (13)
N1	0.0597 (12)	0.0375 (11)	0.0487 (12)	−0.0110 (9)	−0.0022 (9)	−0.0058 (9)
N2	0.0631 (13)	0.0401 (12)	0.0516 (13)	−0.0097 (10)	0.0019 (10)	−0.0092 (9)
N3	0.167 (3)	0.0614 (17)	0.073 (2)	−0.0194 (18)	0.0156 (19)	−0.0031 (15)
O1	0.0984 (15)	0.0522 (12)	0.0589 (12)	−0.0172 (11)	0.0024 (10)	−0.0174 (9)
O2	0.0928 (14)	0.0542 (11)	0.0506 (11)	−0.0238 (10)	0.0010 (9)	−0.0047 (8)
O3	0.136 (2)	0.0370 (11)	0.0682 (14)	−0.0105 (11)	0.0055 (13)	−0.0070 (9)
O4	0.0914 (14)	0.0451 (10)	0.0536 (12)	−0.0108 (10)	0.0064 (10)	−0.0083 (8)

### Geometric parameters (Å, °)

**Table:**

C1—N2	1.344 (3)	C6—O3	1.203 (3)
C1—C2	1.390 (4)	C6—O4	1.320 (3)
C1—C6	1.483 (4)	C7—O4	1.461 (3)
C2—C3	1.378 (3)	C7—H7A	0.9600
C2—H2	0.9300	C7—H7B	0.9600
C3—N1	1.374 (3)	C7—H7C	0.9600
C3—C4	1.472 (4)	C8—C9	1.444 (4)
C4—O1	1.201 (3)	C8—N1	1.464 (3)
C4—O2	1.330 (3)	C8—H8A	0.9700
C5—O2	1.453 (4)	C8—H8B	0.9700
C5—H5A	0.9600	C9—N3	1.139 (4)
C5—H5B	0.9600	N1—N2	1.342 (3)
C5—H5C	0.9600		
			
N2—C1—C2	111.5 (2)	O4—C6—C1	113.0 (2)
N2—C1—C6	121.5 (2)	O4—C7—H7A	109.5
C2—C1—C6	127.0 (2)	O4—C7—H7B	109.5
C3—C2—C1	105.6 (2)	H7A—C7—H7B	109.5
C3—C2—H2	127.2	O4—C7—H7C	109.5
C1—C2—H2	127.2	H7A—C7—H7C	109.5
N1—C3—C2	105.9 (2)	H7B—C7—H7C	109.5
N1—C3—C4	122.6 (2)	C9—C8—N1	111.2 (2)
C2—C3—C4	131.5 (2)	C9—C8—H8A	109.4
O1—C4—O2	124.9 (3)	N1—C8—H8A	109.4
O1—C4—C3	125.2 (2)	C9—C8—H8B	109.4
O2—C4—C3	110.0 (2)	N1—C8—H8B	109.4
O2—C5—H5A	109.5	H8A—C8—H8B	108.0
O2—C5—H5B	109.5	N3—C9—C8	175.4 (3)
H5A—C5—H5B	109.5	N2—N1—C3	112.2 (2)
O2—C5—H5C	109.5	N2—N1—C8	120.3 (2)
H5A—C5—H5C	109.5	C3—N1—C8	127.5 (2)
H5B—C5—H5C	109.5	N1—N2—C1	104.8 (2)
O3—C6—O4	124.8 (3)	C4—O2—C5	116.9 (2)
O3—C6—C1	122.2 (3)	C6—O4—C7	116.4 (2)
			
N2—C1—C2—C3	−0.1 (3)	C4—C3—N1—N2	178.5 (2)
C6—C1—C2—C3	179.7 (3)	C2—C3—N1—C8	178.9 (2)
C1—C2—C3—N1	0.1 (3)	C4—C3—N1—C8	−2.5 (4)
C1—C2—C3—C4	−178.3 (3)	C9—C8—N1—N2	−2.1 (4)
N1—C3—C4—O1	−3.7 (4)	C9—C8—N1—C3	178.9 (3)
C2—C3—C4—O1	174.5 (3)	C3—N1—N2—C1	0.1 (3)
N1—C3—C4—O2	176.9 (2)	C8—N1—N2—C1	−179.0 (2)
C2—C3—C4—O2	−4.8 (4)	C2—C1—N2—N1	0.0 (3)
N2—C1—C6—O3	175.0 (3)	C6—C1—N2—N1	−179.8 (2)
C2—C1—C6—O3	−4.7 (4)	O1—C4—O2—C5	−3.5 (4)
N2—C1—C6—O4	−4.3 (4)	C3—C4—O2—C5	175.9 (2)
C2—C1—C6—O4	176.0 (2)	O3—C6—O4—C7	0.8 (4)
N1—C8—C9—N3	176 (5)	C1—C6—O4—C7	−180.0 (2)
C2—C3—N1—N2	−0.1 (3)		

### Hydrogen-bond geometry (Å, °)

**Table:**

*D*—H···*A*	*D*—H	H···*A*	*D*···*A*	*D*—H···*A*
C2—H2···O3^i^	0.93	2.33	3.256 (4)	176

Symmetry codes: (i) −*x*+1, −*y*, −*z*+2.

### Table 2 π-π interaction (Å, °)

**Table:**

Group 1	Group 2	α	Cg–Cg	τ
Cg1	Cg1^i^	0.03	3.793 (4)	26.14

Symmetry codes: (i) 1-x, 1-y, 2-z. Cg1 is the centroid of ring N1, N2, C1, C2, C3. α is the dihedral angle between the planes τ is the angle subtended by the plane normal to the centroid–centroid vector.
